# Water T2 could predict functional decline in patients with dysferlinopathy

**DOI:** 10.1002/jcsm.13063

**Published:** 2022-09-04

**Authors:** Ursula Moore, Ericky Caldas de Almeida Araújo, Harmen Reyngoudt, Heather Gordish‐Dressman, Fiona E. Smith, Ian Wilson, Meredith James, Anna Mayhew, Laura Rufibach, John W. Day, Kristi J. Jones, Diana X. Bharucha‐Goebel, Emmanuelle Salort‐Campana, Alan Pestronk, Maggie C. Walter, Carmen Paradas, Tanya Stojkovic, Madoka Mori‐Yoshimura, Elena Bravver, Elena Pegoraro, Jerry R. Mendell, The Jain COS Consortium, Kate Bushby, Andrew M. Blamire, Volker Straub, Pierre G. Carlier, Jordi Diaz‐Manera

**Affiliations:** ^1^ The John Walton Muscular Dystrophy Research Centre, Translational and Clinical Research Institute Newcastle University and Newcastle Hospitals NHS Foundation Trust Newcastle upon Tyne UK; ^2^ NMR Laboratory, Neuromuscular Investigation Center Institute of Myology Paris France; ^3^ NMR Laboratory CEA/DRF/IBFJ/MIRCen Paris France; ^4^ Center for Translational Science, Division of Biostatistics and Study Methodology Children's National Health System Washington DC USA; ^5^ Pediatrics, Epidemiology and Biostatistics George Washington University Washington DC USA; ^6^ Magnetic Resonance Centre, Translational and Clinical Research Institute Newcastle University Newcastle upon Tyne UK; ^7^ Jain Foundation Seattle WA USA; ^8^ Department of Neurology and Neurological Sciences Stanford University School of Medicine Stanford CA USA; ^9^ The Children's Hospital at Westmead and The University of Sydney Sydney NSW Australia; ^10^ Department of Neurology Children's National Health System Washington DC USA; ^11^ National Institutes of Health (NINDS) Bethesda MD USA; ^12^ Service des maladies neuromusculaire et de la SLA Hôpital de La Timone Marseille France; ^13^ Department of Neurology Washington University School of Medicine St. Louis MO USA; ^14^ Friedrich‐Baur‐Institute, Department of Neurology Ludwig‐Maximilians‐University of Munich Munich Germany; ^15^ Neuromuscular Unit, Department of Neurology Hospital U. Virgen del Rocío/Instituto de Biomedicina de Sevilla Sevilla Spain; ^16^ Centre de référence des maladies neuromusculaires Institut de Myologie, AP‐HP, Sorbonne Université, Hôpital Pitié‐Salpêtrière Paris France; ^17^ Department of Neurology National Center Hospital, National Center of Neurology and Psychiatry Tokyo Japan; ^18^ Neuroscience Institute Carolinas Neuromuscular/ALS‐MDA Center, Carolinas HealthCare System Charlotte NC USA; ^19^ Department of Neuroscience University of Padova Padua Italy; ^20^ The Abigail Wexner Research Institute at Nationwide Children's Hospital Columbus OH USA; ^21^ Université Paris‐Saclay, CEA, DRF, Service Hospitalier Frederic Joliot Orsay France; ^22^ Neuromuscular Disorders Unit, Neurology Department Hospital de la Santa Creu i Sant Pau Barcelona Spain; ^23^ Centro de Investigación Biomédica en Red en Enfermedades Raras (CIBERER) Madrid Spain

**Keywords:** Magnetic resonance imaging, Water T2, Limb girdle muscular dystrophy, Limb girdle muscular dystrophy R2, Limb girdle muscular dystrophy 2B

## Abstract

**Background:**

Water T2 (T2_H2O_) mapping is increasingly being used in muscular dystrophies to assess active muscle damage. It has been suggested as a surrogate outcome measure for clinical trials. Here, we investigated the prognostic utility of T2_H2O_ to identify changes in muscle function over time in limb girdle muscular dystrophies.

**Methods:**

Patients with genetically confirmed dysferlinopathy were assessed as part of the Jain Foundation Clinical Outcomes Study in dysferlinopathy. The cohort included 18 patients from two sites, both equipped with 3‐tesla magnetic resonance imaging (MRI) systems from the same vendor. T2_H2O_ value was defined as higher or lower than the median in each muscle bilaterally. The degree of deterioration on four functional tests over 3 years was assessed in a linear model against covariates of high or low T2_H2O_ at baseline, age, disease duration, and baseline function.

**Results:**

A higher T2_H2O_ at baseline significantly correlated with a greater decline on functional tests in 21 out of 35 muscles and was never associated with slower decline. Higher baseline T2_H2O_ in adductor magnus, vastus intermedius, vastus lateralis, and vastus medialis were the most sensitive, being associated bilaterally with greater decline in multiple timed tests. Patients with a higher than median baseline T2_H2O_ (>40.6 ms) in the right vastus medialis deteriorated 11 points more on the North Star Ambulatory Assessment for Dysferlinopathy and lost an additional 86 m on the 6‐min walk than those with a lower T2_H2O_ (<40.6 ms). Optimum sensitivity and specificity thresholds for predicting decline were 39.0 ms in adductor magnus and vastus intermedius, 40.0 ms in vastus medialis, and 40.5 ms in vastus lateralis from different sites equipped with different MRI systems.

**Conclusions:**

In dysferlinopathy, T2_H2O_ did not correlate with current functional ability. However, T2_H2O_ at baseline was higher in patients who worsened more rapidly on functional tests. This suggests that inter‐patient differences in functional decline over time may be, in part, explained by different severities of the active muscle damage, assessed by T2_H2O_ measure at baseline. Significant challenges remain in standardizing T2_H2O_ values across sites to allow determining globally applicable thresholds. The results from the present work are encouraging and suggest that T2_H2O_ could be used to improve prognostication, patient selection, and disease modelling for clinical trials.

## Introduction

Predicting functional decline in muscular dystrophies is a difficult task. There are many paediatric and adult onset forms of muscular dystrophy and they display highly variable rates of disease progression, yet few clues to the cause of the variability have been identified.^1^ In some diseases, particularly the more rapidly progressive Duchenne muscular dystrophy (DMD), identifying current functional ability may suggest the next function to be lost, leading to a predictable set of disease milestones.^2^, ^3^ Biomarkers that correlate with current function can therefore predict the next step in disease progression in DMD.^4^ However, in the slowly progressive limb girdle muscular dystrophies (LGMD), predicting short‐term functional changes presents more of a challenge. Dysferlinopathy is a form of LGMD, most commonly described as LGMDR2 or Miyoshi myopathy (MMD1).^5^, ^6^ In this disease, from the same functional starting point, one patient may remain stable for 3 years, while another deteriorates significantly over only 1 year.^7^, ^8^

Being able to predict progression of muscular dystrophies has several advantages. Patients may benefit from clearer expectations about the future and more tailored care. Moreover, for clinical trials aiming to demonstrate an effective intervention, it is important to have a well‐matched cohort, or at least to understand the differences in anticipated disease progression without intervention.^9^, ^10^ As interventional therapies become a reality for the muscular dystrophies, the need to identify biomarkers able to predict upcoming functional decline has intensified.^11^, ^12^

Magnetic resonance imaging (MRI) has been proposed as one such biomarker.^4^, ^11^, ^12^, ^13^ The most used sequences in clinics are both qualitative and include T1‐weighted, which detects fatty replacement of the muscles, and fat‐suppressed T2‐weighted imaging, which signal is increased in several diseases being usually related to oedema and inflammation, although these can be masked by the presence of fatty infiltrations due to the fat suppression.^14^ Although these sequences have been demonstrated to be useful for the diagnosis of patients with neuromuscular diseases, their interpretation can be biased and they lack reproducibility, which hampers their application in longitudinal follow‐up studies designed over short periods of time.^15^ In contrast, quantitative MRI methods such as Dixon‐based fat‐fraction (FF) mapping, which provides an objective measurement of the amount of fat present in the skeletal muscles, have been demonstrated to correlate with muscle function and to capture changes in muscle structure over short periods of time. Such methods are being implemented in natural history studies and also clinical trials.^15^, ^16^, ^17^ However, its role as a predictor of changes in muscle function has not been demonstrated so far.

T2 mapping sequences have also been used to study changes in muscle structure in several neuromuscular diseases (NMDs). Skeletal muscle T2 is elevated in the presence of oedema, inflammation, and necrosis, as a consequence of increased water mobility and disrupted tissue ultrastructure. However, fatty replacement is a common pathway in the disease progression of most NMDs, which also results in increased T2 values, because the T2 of fat is much longer than that of muscle.^15^, ^18^ In order to assess the current status of the residual skeletal muscle tissue, independently from the irreversible end‐stage fatty replacement, water T2 (T2_H2O_) mapping methods have been developed, allowing the detection of inflammatory, oedematous, and necrotic features in fatty‐infiltrated muscles.^19^, ^20^

T2_H2O_ is increased in several NMDs and its capacity to predict changes in muscle structure, mainly the fat replacement that follows muscle fibre loss, has been reported in several diseases.^21^, ^22^, ^23^, ^24^ Recent data from the Clinical Outcomes Study (COS) baseline visit suggest that T2_H2O_ correlates with fatty replacement over time in dysferlinopathy patients, confirming the capacity of T2_H2O_ to identify active damage leading to muscle fibre loss and expansion of fat tissue.^25^ However, the capacity of T2_H2O_ to identify patients who will experience a quicker and more severe clinical progression over a short period of time remains to be demonstrated. If it could be shown to predict progression, T2_H2O_ could be used both in clinics to identify patients at risk of progression and also in clinical trials to select patients who may deteriorate over a short period of time and whose results could be more informative about the effectivity of experimental therapies.

We hypothesized that inter‐patient variation in active muscle damage, assessed by T2_H2O_, may underlie and predict subsequent differences in disease progression from the same functional starting point in patients with dysferlinopathy. In this paper, we assess a series of quantitative MRI parameters, including FF, contractile cross‐sectional area (cCSA), and T2_H2O_ value, in each of the lower limb muscles and how they relate to subsequent disease progression in a range of functional and timed tests in patients with dysferlinopathy, to determine whether any of these parameters can be effectively used to predict muscle function loss.

## Methods

### Patients

Patients were selected from the Jain Foundation COS in dysferlinopathy.^26^ COS is an international, multicentric, prospective natural history study involving 15 centres in the USA, Europe, Asia, and Oceania. COS recruited and assessed 182 patients over a 3‐year period. Assessments performed included medical, physical therapist, biochemical, cardiovascular, and respiratory and MRI that were performed at baseline visit and then every year until Year 3. All patients had a diagnosis of dysferlinopathy, confirmed by genetic testing, with two pathogenic mutations or one pathogenic mutation and evidence of reduced dysferlin expression on a western blot of skeletal muscle and/or monocytes. The baseline, 1‐year data, and 3‐year functional progression and quantitative muscle MRI analysis have previously been published.^7^, ^8^, ^25^

**Figure 1:**
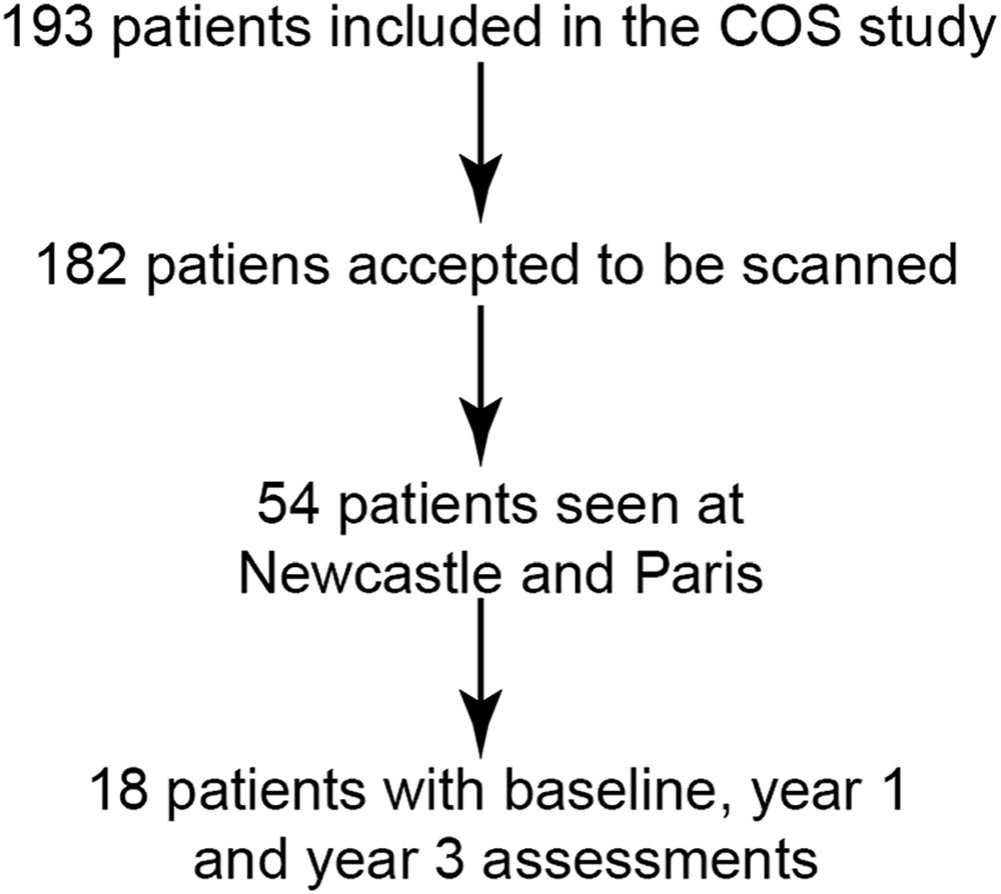
Patients included in this study from the original COS study. COS, Clinical Outcomes Study.

We selected a cohort of patients seen at Newcastle and Paris to limit the impact of inter‐site variability. These two sites had the largest number of patients with MRI results and have previously been shown to have high inter‐site reliability. In order to identify if quantitative MRI measurement could predict changes in muscle function, we only included ambulant patients who completed a set of four functional assessments including the North Star Assessment for limb girdle‐type muscular dystrophies [North Star Ambulatory Assessment for Dysferlinopathy (NSAD)], the 6‐min walk test (smwt), the timed up and go (TUG) test, and the 10‐m walk test (10MWT) at baseline, Year 1, and Year 3. The cohort included a total of 18 patients who had an NSAD score of more than 15 points (*Figure* 1).

### Quantitative muscle magnetic resonance imaging: acquisition and processing of Dixon and T2_H2O_ imaging data

Patients were imaged using 3.0‐tesla MRI clinical scanners from two different vendors: Newcastle (Philips) and Paris (Siemens). MRI acquisition parameters were standardized across sites before the start of the study. Patients were positioned feet‐first supine, and all MRI sequences were centred at one‐third of the femur from the superior border of the patella and at the widest part of the calf. The acquisition protocol for FF and T2_H2O_ mapping in the COS study has been recently reported.^25^

Water and fat images were reconstructed using in‐house Matlab code (MathWorks, Natick, MA, USA), which incorporated hierarchical IDEAL (iterative decomposition of water and fat with echo asymmetry and least‐squares estimation) and the Tsao–Jiang algorithm for separating multiple chemical species by hierarchical decomposition and direct estimation of phase.^27^ Using the mean‐square error (MSE) images, quantitative T2_H2O_ maps were reconstructed based on a tri‐exponential fitting procedure.^19^, ^21^ Regions of interest (ROIs) were drawn manually using a free software tool (https://www.itksnap.org) in the same five central slices on the shortest echo time (TE) image of the MSE series images by a single investigator as has been already described.^25^ ROIs were drawn on both the left and right sides for seven leg muscles (extensor digitorum, tibialis anterior, tibialis posterior, peroneus longus, soleus, gastrocnemius medialis, and gastrocnemius lateralis) and in nine thigh muscles (vastus lateralis, vastus intermedius, vastus medialis, gracilis, sartorius, adductor magnus, biceps femoris long head, semimembranosus, and semitendinosus). For the determination of FF and cross‐sectional area (CSA), the boundaries of the ROIs were drawn following individual muscle delineation, avoiding inclusion of other muscles, subcutaneous and intermuscular fat, tendons, and major blood vessels. cCSA was calculated for each muscle using cCSA = (1 − FF_mean_) * CSA. For the assessment of T2_H2O_, ROIs delineated the interior of the muscle, avoiding visible fasciae and blood vessels. ROIs that included <10 pixels were excluded for analysis.

After quality control, FF and cCSA calculation was not possible in all slices for all patients. This resulted in some slices for which there was a T2_H2O_ value but not an FF and a cCSA value. Assessment of correlations between T2_H2O_ and FF and cCSA used only those data sets where all variables were available (*n* numbers shown in *Table* 1).

**Table 1:** Correlation between T2_H2O_ and changes in muscle function, fat fraction, and contractile cross‐sectional area over 3 years

Muscle [left (L)/right (R)]	Median T2_H2O_ value (range)	ΔNSAD	Δ6MWT velocity in m/s	Δ6MWT distance in m	ΔTUG rate (event/s)	Δ10MWT velocity (m/s)	*N* with Dixon data	ΔFF (%)	ΔcCSA (mm^2^)
**Vastus intermedius (L)**	40.0 (34.3–46.4	**−8** *	**−0.18** *	**−64.8** *	**−0.04** **	**−0.06** *	16	9.4	−54.7
**Vastus intermedius (R)**	40.6 (35.8–47.2)	**−11** **	−0.14	−50.4	**−0.04** **	**−0.06** **	16	7.8	−181.1
**Vastus lateralis (L)**	41.4 (27.3–48.0)	**−9** **	−0.10	−36.0	**−0.03** *	**−0.05** *	16	8.4	−551.0
**Vastus lateralis (R)**	42.7 (35.2–51.4)	−6	−0.08	−28.8	**−0.03** *	**−0.05** *	16	**10.3** *	−606.1
**Vastus medialis (L)**	39.5 (26.9–50.1)	**−8** *	**−0.20** *	**−72.0** *	−0.02	**−0.05** *	16	**9.8** **	−315.9
**Vastus medialis (R)**	42.0 (33.1–48.8)	**−11** **	**−0.24** **	**−86.4** **	**−0.04** ***	**−0.07** **	15	**9.7** **	−441.4
**Adductor magnus (L)**	39.3 (33.1–45.8)	**−7** *	−0.14	−50.4	**−0.03** **	−0.04	16	15.5	−40.7
**Adductor magnus (R)**	39.0 (33.6–47.2	**−10** **	**−0.18** *	**−64.8** *	**−0.04** ***	**−0.07** **	16	13.3	−156.9
Gracilis (L)	38.5 (33.4–43.0)	−4	−0.15	−54.0	**−0.03** **	−0.03	16	9.1	−123.6
Gracilis (R)	37.5 (33.0–44.3)	−6	**−0.17** *	**−61.2** *	**−0.03** **	−0.03	16	3.5	−141.9
Sartorius (L)	38.4 (32.0–43.9)	−4	**−0.16** *	**−57.6** *	**−0.03** **	0	16	12.0	−142.6
Sartorius (R)	39.3 (34.5–44.8)	**−8** **	−0.11	−39.6	**−0.03** **	−0.03	16	7.9	−88.1
Biceps femoris (L)	39.6 (26.4–51.5)	−2	0.05	−18.0	−0.02	0.02	15	5.9	−162.9
Biceps femoris (R)	38.7 (27.7–50.4)	**−9** **	−0.16	−57.6	**−0.03** *	−0.02	14	13.6	−181.4
Semimembranosus (L)	38.3 (25.7–47.8)	**−7** *	−0.13	−46.8	−0.03	−0.02	13	13.1	−113.1
Semimembranosus (R)	38.1 (20.3–46.2)	−3	−0.08	−28.8	−0.01	0.02	13	**10.0** *	−168.7
Semitendinosus (L)	36.7 (28.8–44.6)	−5	−0.01	−3.6	−0.01	−0.01	16	7.1	−57.8
Semitendinosus (R)	34.8 (22.6–47)	−4	−0.09	−32.4	−0.01	0.01	16	9.4	−35.8
Tibialis anterior (L)	39.4 (23.9–49.4)	**−7** *	−0.14	−50.4	−0.02	0	15	4.0	−41.1
Tibialis anterior (R)	38.6 (31.1–53.3)	−2	−0.04	−14.4	−0.01	0	15	−2.4	21.0
Extensor digitorum (L)	40.5 (34.2–50)	**−8** *	−0.11	−39.6	**−0.03** **	−0.01	15	**7.7** *	−50.1
Extensor digitorum (R)	39.6 (34.8–48)	−2	−0.03	−10.8	−0.02	0.01	15	11.3	−95.3
Peroneus (L)	37.1 (23.9–50)	−2	−0.12	−43.2	−0.01	0.01	15	14.5	−159.6
Peroneus (R)	36.4 (25.2–48.2)	−3	−0.11	−39.6	0.02	−0.02	14	13.2	−184.5
Tibialis posterior (L)	40.2 (34.1–50.3)	−2	−0.11	−39.6	**−0.03** *	0.01	15	6.9	−133.8
Tibialis posterior (R)	39.9 (34.8–48.3)	−4	−0.06	−21.6	−0.02	−0.02	15	8.4	−129.9
Soleus (L)	37.3 (23.6–50.4)	−1	−0.09	−32.4	−0.02	0.01	14	6.1	−14.9
Soleus (R)	38.1 (26.9–49.4)	**−9** *	**−0.26** **	**−93.6** **	**−0.04** **	−0.03	15	10.9	−40.5
Gastrocnemius lateralis (L)	36.9 (25.8–46.2)	5	0.03	10.8	0.01	0.02	14	14.1	−151.1
Gastrocnemius lateralis (R)	37.7 (23.1–47.6)	1	−0.06	−21.6	0	0	13	15.8	−34.0
Gastrocnemius medialis (L)	35.9 (26.2–57)	3	0.07	25.2	0.02	**0.05** *	14	3.8	−95.1
Gastrocnemius medialis (R)	38.5 (26.1–60.7)	−4	−0.06	−21.6	−0.02	0	14	0.5	−73.8

*N*, number; Δ6MWT, change in 6‐min walk test between Year 3 and baseline; Δ10MWT, change in 10‐m walk test between Year 3 and baseline; ΔcCSA, change in contractile cross‐sectional area between Year 3 and baseline; ΔFF, change in fat fraction between Year 3 and baseline; ΔNSAD, change in North Star Ambulatory Assessment for Dysferlinopathy between Year 3 and baseline; ΔTUG, change in timed up and go test between Year 3 and baseline.

The table shows mean changes in muscle function tests and their association with high T2_H2O_. Regression coefficients in bold were statistically significant at the 0.05 level.

* *P* < 0.05.

** *P* < 0.01.

*** *P* < 0.001.

### Data analysis

#### Higher or lower than median T2_H2O_

The median T2_H2O_ of each muscle in each patient was categorized as either (a) greater than or equal to the cohort median T2_H2O_ value for that muscle (high T2_H2O_) or (b) less than the cohort median T2_H2O_ value for that muscle (low T2_H2O_). The change in functional score (ΔNSAD) or change in velocity in the timed tests (Δsmwt, ΔTUG, and Δ10MWT) over 3 years was calculated using assessments from baseline and Year 3.

Demographic information was reviewed, including age, disease duration, and baseline functional assessment score. Demographic data and baseline functional assessment results were compared between high and low T2_H2O_ values in each muscle. Median values were compared between groups using a Mann–Whitney test.

For each functional assessment, a linear model of the change in functional score over 3 years was performed with high or low baseline T2_H2O_ as a predictor and disease duration, age, and baseline functional score as covariates. Timed tests were assessed as velocities to give normally distributed values. This model produces an estimate of the additional change in functional score seen in those with a high T2_H2O_ compared with those with a low T2_H2O_ value, when disease duration, age, and baseline functional score are held constant. The analysis was performed for each functional assessment using the T2_H2O_ value from each of the 16 muscles on each side in turn (4 functional assessments × 32 muscles—left and right). This method was repeated with FF and cCSA data. For each muscle, the change in FF and cCSA over 3 years was modelled against the baseline T2_H2O_ value, with disease duration, age, and baseline FF and cCSA as covariates.

Differences were considered significant if the *P*‐value was lower than 0.05. The *P*‐values were adjusted using the Holm–Bonferroni correction to assess statistical significance in the presence of multiple comparisons.

#### Identifying a T2_H2O_ threshold that identifies higher changes in North Star Ambulatory Assessment for Dysferlinopathy than expected

Previous studies modelling the COS cohort of patients over time have demonstrated that NSAD score declines by 1.68 points per year, and therefore, over 3 years, a decline of more than 5.04 points identifies those with faster than average functional decline.^8^ Each patient was classed as having a decline of more than 5 points in NSAD score (positive result) or <5 points (negative result). We calculated the cut‐off point in T2_H2O_ with the higher sensitivity and specificity to distinguish between patients progressing more and less than 5 points in NSAD using receiver operating characteristic curves (ROC curves) using R software (https://www.r‐project.org). We included in this analysis only those muscles whose baseline T2_H2O_ correlated with changes in muscle function tests from baseline to the last visit assessment.

## Results

### Demographics

The cohort consisted of 18 ambulant patients (7 male) assessed in Newcastle (12 patients) or Paris (6 patients). Patients had a median age of 32.5 years (range 19–71 years) and had had symptoms for a median of 11 years (range 3–22 years). Demographic information split by T2_H2O_ value per muscle is listed in Supporting Information, *Table* *S1*.

### T2_H2O_ values vary between muscles

Median T2_H2O_ values varied from a minimum of 35.9 ms in gastrocnemius medialis (left) to a maximum of 42.7 ms in vastus lateralis (right) (*Table* 1). Within each muscle, T2_H2O_ was not significantly different between left and right (Wilcoxon's test). T2_H2O_ did not correlate with functional score, FF, or cCSA at baseline (*Table* *S2*).

### High T2_H2O_ value was significantly correlated with functional deterioration

Median T2_H2O_ values at baseline significantly correlated with a worsening for at least one functional assessment over the next 3 years in 19 of the 32 muscles studied (*Table* 1). Muscles with high T2_H2O_ were always associated with faster functional deterioration.

High T2_H2O_ in adductor magnus and vastus intermedius/lateralis/medialis muscles stood out as being consistently and bilaterally significantly correlated with functional progression, demonstrating a greater deterioration in at least two functional assessments bilaterally (*Table* 1).

In vastus lateralis (bilateral), vastus medialis (right), semimembranosus (left), and extensor digitorum (left), median T2_H2O_ values significantly correlated with an increase in FF over 3 years. Median T2_H2O_ values did not correlate with a change in cCSA bilaterally in any muscle after 3 years of follow‐up (*Table* 1).

### Determining a T2_H2O_ value for prediction

**Figure 2:**
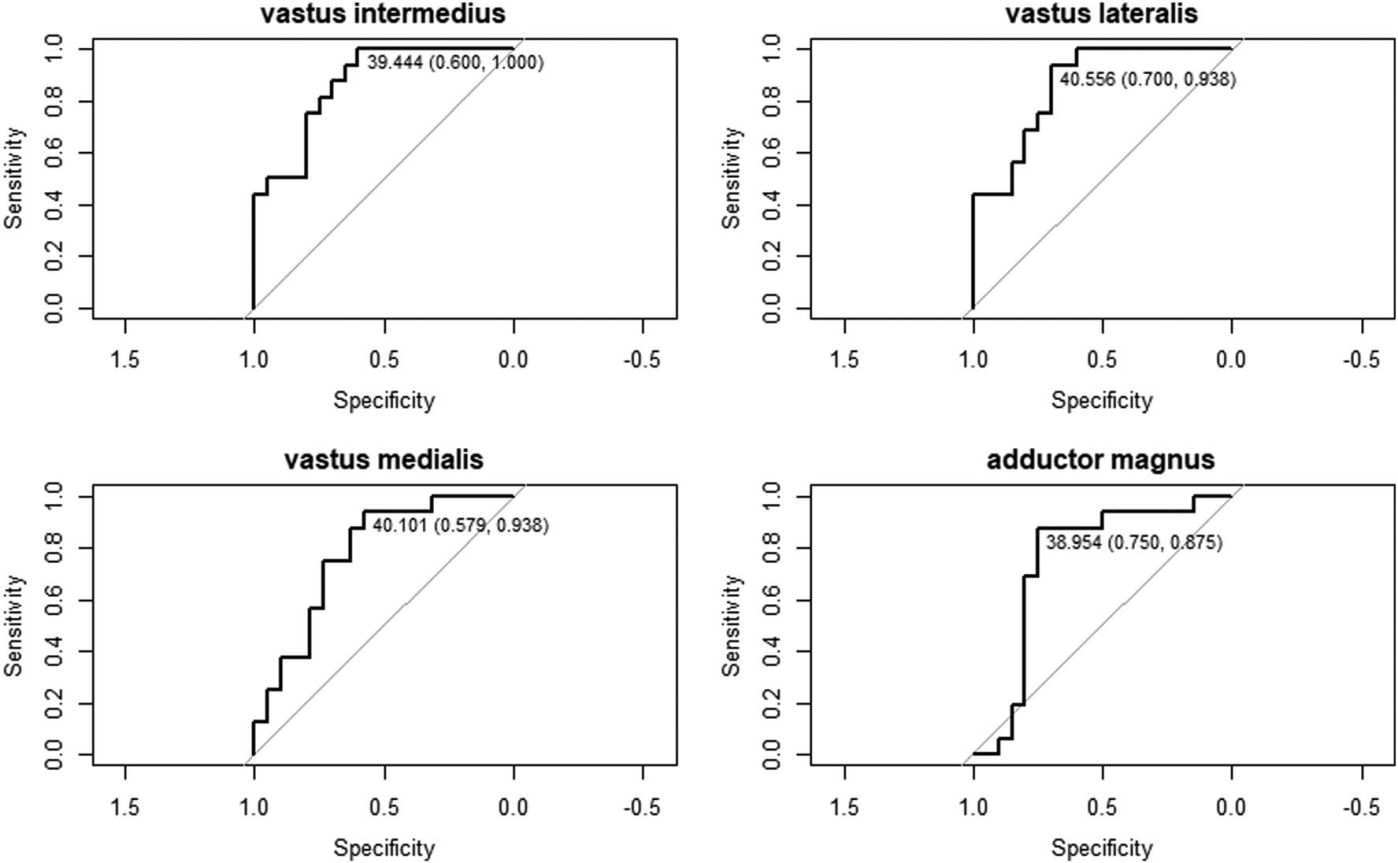
Receiver operating characteristic curve of the sensitivity and specificity achieved by all possible T2_H2O_ relaxation times. The area under the curve is listed for each plot. In each curve, the threshold with the highest specificity (with zero false positives) and the threshold with optimum sensitivity and specificity are listed.

We studied the T2_H2O_ value thresholds that maximized sensitivity and specificity for predicting decline in function using ROC curves of the adductor magnus, vastus intermedius, vastus lateralis, and vastus medialis. The T2_H2O_ value thresholds obtained were 39.0 ms [88% sensitive and 75% specific, area under the curve (AUC) 0.847 (0.634–1)] in adductor magnus, 39.4 ms [100% sensitive, 60% specific, AUC 0.917 (0.782–1)] in vastus intermedius, 40.5 ms [94% sensitive, 70% specific, AUC 0.889 (0.734–1)] in vastus lateralis, and 40.1 ms [94% sensitive, 40% specific, AUC 0.875 (0.699–1)] in vastus medialis (*Figure* 2).

### Validating T2_H2O_ thresholds

**Figure 3:**
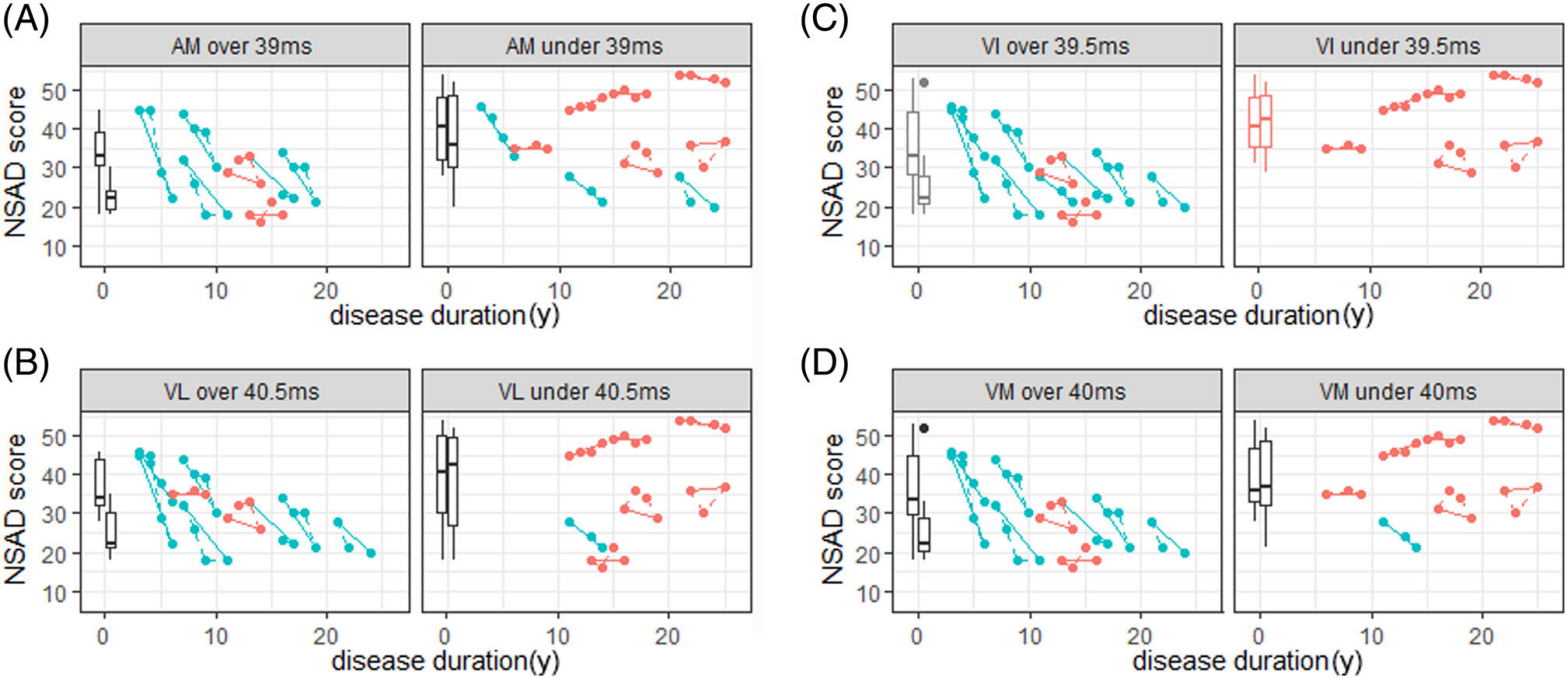
Disease progression in the cohort analysed, grouped by T2_H2O_ threshold value identified for *(A)* adductor magnus (AM), *(B)* vastus intermedius (VI), *(C)* vastus lateralis (VL), and *(D)* vastus medialis (VM). Dots showing the North Star Ambulatory Assessment for Dysferlinopathy (NSAD) score at each time point are grouped by dashed lines to illustrate individual patient trajectories on the NSAD score over 3 years. Those deteriorating more than 5 points over 3 years are coloured blue, and those deteriorating 5 or less points are coloured red. Box plots represent the median, interquartile range, and range of NSAD score for patients at baseline (left) and Year 3 (right).

Seventeen of the 18 patients had bilateral T2_H2O_ results for adductor magnus, vastus intermedius, vastus lateralis, and vastus medialis and could be included in this analysis. The sensitivity of predicting decline on the NSAD score with the T2_H2O_ thresholds over both 1 and 3 years is shown in *Table* 2 and *Figure* 3. As shown in *Table* 2, sensitivity and specificity of the measures suggested that those with high T2_H2O_ values were generally rapid progressors. Sensitivity and specificity values weere not improved by requiring all four muscles to be over their indiviudal thresholds or requring only one of the four muscles to be over their individual theshold (*Table* 2).

**Table 2:** Sensitivity and specificity of a T2_H2O_ threshold in predicting decline in North Star Ambulatory Assessment for Dysferlinopathy (NSAD) score

Muscle	Threshold	Analysis	Decline of >1 point on NSAD in 1 year	Decline of >5 points on NSAD in 3 years
Adductor magnus	39.0 ms	Sensitivity	57%	63%
		Specificity	71%	78%
Vastus intermedius	39.4 ms	Sensitivity	86%	100%
		Specificity	57%	67%
Vastus lateralis	40.5 ms	Sensitivity	71%	88%
		Specificity	71%	78%
Vastus medialis	40.1 ms	Sensitivity	86%	88%
		Specificity	57%	67%
**Muscle grouping**				
All four muscles over threshold bilaterally	Respective threshold for each muscle as above	Sensitivity	43%	63%
		Specificity	71%	89%
At least one muscle over threshold bilaterally		Sensitivity	86%	100%
		Specificity	57%	56%

## Discussion

We have shown that a higher T2_H2O_ in some muscles of the lower limbs might be associated with greater functional deterioration in patients with dysferlinopathy. These findings could be particularly helpful in advising patients about prognosis, in selecting patients for clinical trials, and, potentially, as an outcome measure for interventional trials.

There is a large body of evidence already published demonstrating that the amount of fat present in the skeletal muscle, which can be quantified using quantitative MRI (Dixon method) or proton magnetic resonance spectroscopy (^1^H MRS), correlates with muscle function tests and is sensitive to change over short periods of time.^17^, ^28^ However, little is known about the relation of T2_H2O_, which may be an indicator of active muscle damage and muscle function. It has been observed that in dysferlinopathy, the T2_H2O_ does not correlate with baseline functional scores.^25^ T2_H2O_ has been shown to be impacted by high levels of FF (i.e. >60%) as also described in dysferlinopathy.^25^ The data described in the current study, however, were acquired in exclusively ambulant patients, where FF values were predominantly lower than 60%. This suggests that T2_H2O_ could be compared across patients at different stages of muscle loss to offer a snapshot of the current level of disease activity.

Higher than threshold baseline T2_H2O_ value predicted greater decline over both 1 and 3 years, being consistently more accurate over the 3‐year window. This suggests that change in function is a delayed downstream consequence of higher active muscle damage as quantified by the T2_H2O_. This also reflects the slowly progressive nature of the disease, which results generally in small changes in function over 1 year but more consistently over a longer period of 3 years of follow‐up. Despite this, even over a 1‐year period, T2_H2O_ thresholds were able to predict functional decline, which could make T2_H2O_ particularly useful for detecting patients that will probably progress significantly during a clinical trial with a shorter running time.

We found a correlation between T2_H2O_ at baseline and changes in FF in some of the investigated muscles, including the vastus medialis and the vastus intermedius. Correlation between muscle histopathology and quantitative MRI in patients with muscular dystrophy demonstrates histopathological features of inflammation appearing in the muscle, detectable by T2_H2O_, before fat replacement begins.^29^ In our population, it appears that active muscle damage, detected by higher T2_H2O_, correlated with a functional decline in some of the muscles affected. Given the progressive nature of dysferlinopathy, we may anticipate that with a longer period of follow‐up, this increased disease activity would ultimately translate into irreversibly increased FF and loss of cCSA. These findings suggest that T2_H2O_ is a useful measure to demonstrate active muscle damage in remaining muscle in dysferlinopathy and to provide an accurate quantification of potentially salvageable tissue. This could be useful for monitoring response to future treatments, with T2_H2O_ forming a potential early biomarker of treatment efficacy.^30^

However, not all the muscles imaged showed an association between higher T2_H2O_ value and risk of more severe disease progression. The muscles in which T2_H2O_ did not correlate with functional decline were those of the posterior compartment of the thigh and muscles of the leg, apart from the soleus. There are several possible reasons for this:

1. Due to the functional outcome measures chosen: The distal muscle that did show involvement was the soleus, which is heavily involved in walking, while the gastrocnemius and peroneus are less involved when walking on an even surface.^31^ Regarding the posterior compartment of the thigh, walking can be maintained, even in the presence of significant weakness, by using compensatory mechanisms, which may mean that active muscle damage in these muscles does not translate to impairment on these specific outcome measures.^32^
2. Due to a lower degree of active muscle damage in these muscles at the time of assessment: The pattern of muscle involvement in dysferlinopathy includes an early and severe involvement of the soleus, both the gastrocnemius and the peroneus.^13^ The first functional ability to be lost is usually the ability to stand on tiptoes, a function highly reliant on the gastrocnemius and soleus muscles.^33^ The T2_H2O_ values seen in these muscles were lower than those in the vasti muscles, and these muscles showed a higher fat replacement by the time of assessment as they are affected very early during the disease's progression.^13^
3. Due to the higher FF in these muscles: FFs in the posterior compartment of the thigh and the gastrocnemius were among the highest of all the muscles assessed (*Table* *S2*). T2_H2O_ is much more heterogeneous in more fatty‐replaced muscles, as has been shown.^34^ In this sense, investigation in nine patients with more advanced dysferlinopathy and higher FFs found lower T2_H2O_ in patients compared with healthy controls.^35^ These data suggest that at those stages, T2_H2O_ would probably not be useful to identify active muscle damage due to the massive replacement by fat.^34^

The association of T2_H2O_ with functional test outcomes in specific muscles demonstrates the importance of selecting an appropriate ‘reporter’ muscle that is related to the functional outcome of interest and stage of disease progression. In this analysis, the muscles most associated with the timed tests and NSAD score were the vasti and the adductor magnus muscles. However, we may also anticipate that gluteal muscles, hip extensors, and abductors, which are important in rising from the floor and walking, may constitute useful reporter muscles.^31^ Unfortunately, the COS study did not include T2_H2O_ data from the gluteal muscles and so we were unable to investigate this further. As disease progresses, useful reporter muscles may change to those with lower FFs. In non‐ambulant patients, where outcome measures focus more on the upper limbs, which are generally preserved for longer in dysferlinopathy, one of the arm muscles may be a better reporter muscle.^10^, ^13^ We hope to address this in future, after completion of the clinical outcome extension study, which includes upper limb MRI.

We felt that it would be clinically useful for prognostication or trial cohort selection if a specific threshold could be defined above which T2_H2O_ is considered ‘high’ and patients may be expected to progress more rapidly. We tried to validate these thresholds in a second cohort of dysferlin patients participating in COS study that were scanned in 3‐ and 1.5‐tesla scanners at six other sites, but even though higher T2_H2O_ levels were associated with faster progression, the accuracy, sensitivity, and specificity were lower as it is shown in the supporting information. There are many reasons that could explain these results including, among others, the different field strengths, which is known to affect the T2; the different vendor‐specific sequence details such as radio frequency pulses and crusher‐gradient schemes, which impact the measured magnetic resonance (MR) signal and hence the observed T2; and the transmitter and receiver coils that vary between vendors and even between system versions from the same vendor, which also impact the measured MR signal. While the scan protocols were standardized and the same post‐processing methodology was used for all T2_H2O_ data and was conducted by the same team, normalization across sites by means of control data from the same volunteers in all sites was not practicable.^15^, ^16^ We also attempted to correct for the difference in field strength by applying a correction factor based on published data to the results acquired on 1.5‐tesla scanners.^36^ However, this scaling factor is only an approximation that has been obtained in a few number of healthy controls and likely still leaves significant inter‐scanner variability reinforcing the need of further research in this specific topic. In addition, besides hardware‐related differences that affect T2, variations in patient status, such as recent exercise activity, are known to impact on the observed T2_H2O_.^15^ In this regard, patients participating in the COS study were imaged before physio assessments were performed and were asked to avoid doing any exercise the week before the visit. Most of the patients used a taxi to go to the hospital or imaging centre, in order to reduce the impact that the exercise could have in the MRI values, and were also asked to lie down before being scanned. The data obtained in our extension cohort (supporting information results) unfortunately do not allow us to take any final conclusion about what could be the causes of the lack of validation of the thresholds obtained, indicating that more research is needed in this topic. In this sense, we think that clinical or clinical trial‐based applications attempting to use T2_H2O_ for predicting disease progression across multiple sites would require additional research to identify the optimum standardization of scan protocol, T2_H2O_ mapping, and normalization of data between sites before starting the trial.^37^

This study was conducted in patients with dysferlinopathy. However, the findings may be applicable to other slowly progressive forms of muscular dystrophy, which show periods of both deterioration and stability. Muscular dystrophies share a common pathomechanism of repeated cycles of muscle damage and attempted repair, with release of pro‐inflammatory cytokines and immune cell influx.^38^, ^39^, ^40^, ^41^ Such patterns of inflammation and oedema are detected by T2_H2O_, suggesting that our finding may be extrapolated to other muscular dystrophies, although this needs to be confirmed in future studies.^29^, ^37^

The results presented in this paper suggest that faster functional disease progression in dysferlinopathy can be predicted by higher T2_H2O_ values in muscles of the thigh, over 1 and 3 years. With further research, we predict that similar patterns would be demonstrated in other slowly progressive forms of muscular dystrophy. However, it is important to take into account that an effort in standardizing the measurements between centres is crucial if these observations are to be employed in clinical trial design and prognostication for patients with muscular dystrophy.

## Conflict of interest

Ursula Moore, Ericky Caldas de Almeida Araujo, Harmen Reyngoudt, Heather Gordish‐Dressman, Fiona E. Smith, Ian Wilson, Meredith James, Anna Mayhew, Jown W. Day, Kristi J. Jones, Diana X. Bhraucha Goebel, Emmanuelle Salort Campana, Alan Pestronk, Maggie Walter, Carmen Paradas, Tanya Stojkovic, Madoka Mori‐Yoshimura, Elene Bravver, Elena Pegoraro, Jerry R. Mendel, Kate Bushby, Andrew M. Blamire, Volker Straub, Pierre Carlier, and Jordi Díaz‐Manera have received funding for research from the Jain Foundation during the conduct of the study.

Laura Rufibach is hired by the Jain Foundation.

## Supporting information


**Figure S1:** **Illustrative scheme describing the tri‐exponential fitting method for T2**_**H2O**_ **mapping applied in the present work.**
In (A) are raw T2‐weighted MSE images at different TEs; The blue, red and black filled circles are placed in subcutaneous‐fat, spared muscle and highly fatty‐replaced muscle. Notice how the signal from spared muscle vanishes much faster than the signal from fat as TE evolves, due to its shorter T2 relaxation time. In (B) are the corresponding theoretically predicted signal evolutions; water signal is assumed to be mono‐exponential, while the fat signal, previously calibrated in the subcutaneous fat of healthy subjects, is described with a bi‐exponential model with known fixed T2 values and corresponding relative fractions. The mixed signal model is fitted to the actual data at each pixel by adjusting the relative water and fat signal fractions at TE = 0, and the T2 value characterizing the water signal.

**Figure S2:** **Disease progression in the original and extension cohorts**, grouped by T2_H2O_ threshold value identified for **A** adductor magnus (AM), **B** vastus intermedius (VI), **C** vastus lateralis (VL) and **D** vastus medialis (VM). Dots showing the NSAD score at each time point are grouped by dashed lines to illustrate individual patient trajectories on the NSAD score over 3 years. Those deteriorating more than 5 points over 3 years are coloured blue, and those deteriorating 5 or less points are coloured red. Box plots represent the median, interquartile range (IQR) and range of NSAD score for patients in that panel at baseline (left) and year 3 (right).

**Table S1:** **Median T2 water value of each muscle analysed in the cohort**
Median T2 water value obtained on each muscle is shown as well as number of patients on each subgroup (higher or lower values than median), the median symptom duration and the median age of each group. L: left. R: Right

**Table S2:** **Results of functional tests in patients with higher or lower than median T2 water value**
The table shows the results of the muscle function tests in the patients that were included in the higher or lower than median T2 water value on each muscle. N: number, smwt: 6 minutes walking test, m/s: meters per second, TTRW: time to run/walk 10 meters, TTUG: time to up&go test,

**Table S3:** Sensitivity and specificity of a T2_H2O_ threshold in predicting decline in NSAD score in the initial and extension cohort

**Data S1.** Supporting Information
