# Supplementary figures and images for: Water T2 could predict functional decline in patients with dysferlinopathy
Source: J Cachexia Sarcopenia Muscle. 2022 Sep 4;13(6):2888–97. doi: 10.1002/jcsm.13063 (PMC9745487; doi:10.1002/jcsm.13063)

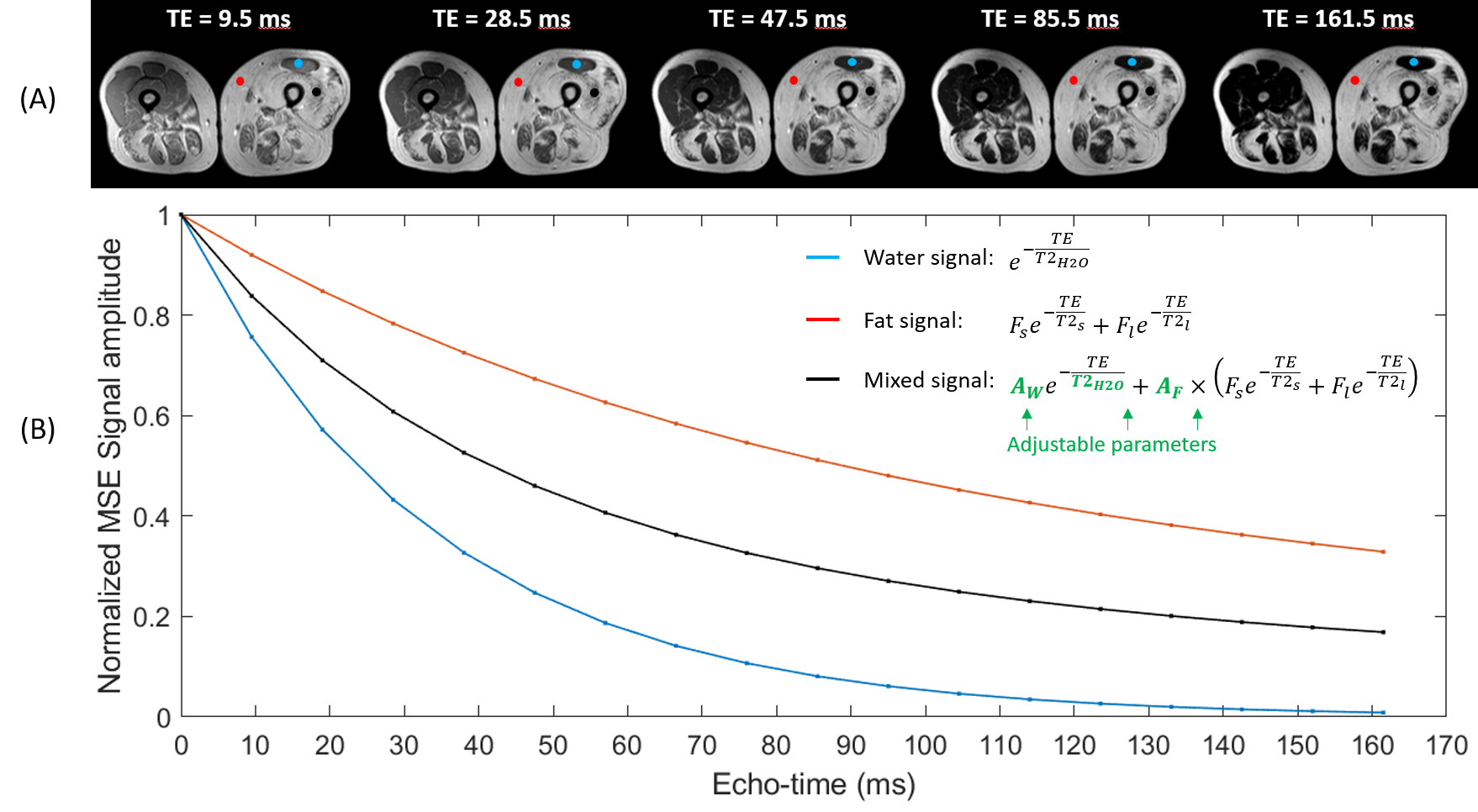

Supplement: Supplementary file 1 — Figure S1: Illustrative scheme describing the tri‐exponential fitting method for T2 H2O mapping applied in the present work. In (A) are raw T2‐weighted MSE images at different TEs; The blue, red and black filled circles are placed in subcutaneous‐fat, spared muscle and highly fatty‐replaced muscle. Notice how the signal from spared muscle vanishes much faster than the signal from fat as TE evolves, due to its shorter T2 relaxation time. In (B) are the corresponding theoretically predicted signal evolutions; water signal is assumed to be mono‐exponential, while the fat signal, previously calibrated in the subcutaneous fat of healthy subjects, is described with a bi‐exponential model with known fixed T2 values and corresponding relative fractions. The mixed signal model is fitted to the actual data at each pixel by adjusting the relative water and fat signal fractions at TE = 0, and the T2 value characterizing the water signal. [file JCSM-13-2888-s006.jpg]

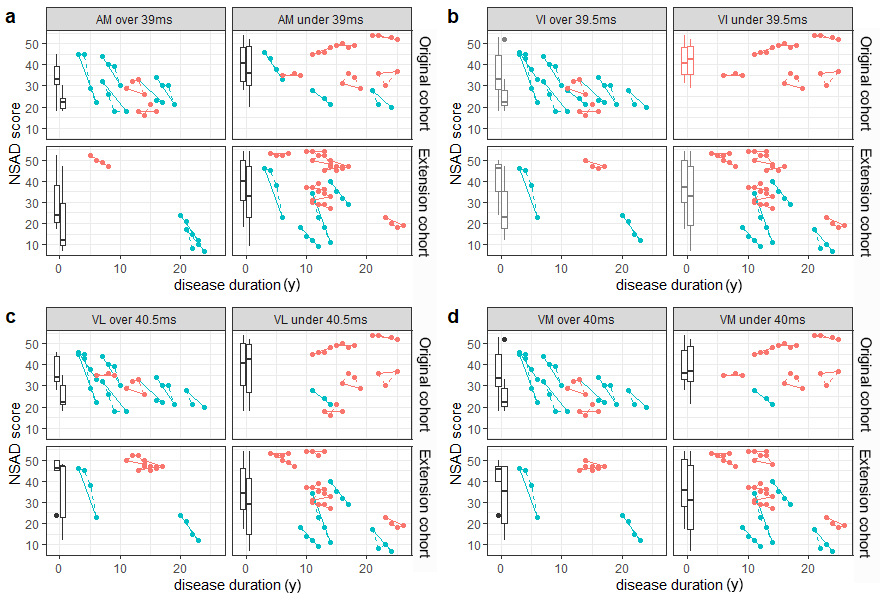

Supplement: Supplementary file 2 — Figure S2: Disease progression in the original and extension cohorts, grouped by T2H2O threshold value identified for A adductor magnus (AM), B vastus intermedius (VI), C vastus lateralis (VL) and D vastus medialis (VM). Dots showing the NSAD score at each time point are grouped by dashed lines to illustrate individual patient trajectories on the NSAD score over 3 years. Those deteriorating more than 5 points over 3 years are coloured blue, and those deteriorating 5 or less points are coloured red. Box plots represent the median, interquartile range (IQR) and range of NSAD score for patients in that panel at baseline (left) and year 3 (right). [file JCSM-13-2888-s004.tif]
