# Supplementary material for: Water T2 could predict functional decline in patients with dysferlinopathy
Source: J Cachexia Sarcopenia Muscle. 2022 Sep 4;13(6):2888–97. doi: 10.1002/jcsm.13063 (PMC9745487; doi:10.1002/jcsm.13063)
Supplement: Supplementary file 3 — Table S1: Median T2 water value of each muscle analysed in the cohort Median T2 water value obtained on each muscle is shown as well as number of patients on each subgroup (higher or lower values than median), the median symptom duration and the median age of each group. L: left. R: Right [file JCSM-13-2888-s003.docx]

| muscle | median water T2 value | number (male) | | Median symptom duration (years) | | Median age (years) | |
| --- | --- | --- | --- | --- | --- | --- | --- |
|  |  | high T2 | low T2 | high T2 | low T2 | high T2 | low T2 |
| Adductor longus (L) | 38.49 | 7(4) | 6(2) | 11 | 14 | 32 | 32.5 |
| Adductor longus (R) | 39.86 | 9(3) | 8(3) | 11 | 11 | 35 | 30.5 |
| Adductor magnus (L) | 39.37 | 9(6) | 9(1) | 11 | 13 | 33 | 32 |
| Adductor magnus (R) | 39.00 | 9(4) | 9(3) | 11 | 15.5 | 29 | 35 |
| Biceps femoris (L) | 39.64 | 8(3) | 9(3) | 11 | 14.5 | 32 | 35 |
| Biceps femoris (R) | 38.76 | 8(2) | 8(4) | 10 | 11 | 33.5 | 34.5 |
| Extensor digitorum (L) | 40.53 | 10(3) | 8(4) | 11 | 14.5 | 29 | 38.5 |
| Extensor digitorum (R) | 39.64 | 9(3) | 9(4) | 11 | 12 | 32 | 35 |
| Gastrocnemius lateralis (L) | 36.90 | 8(1) | 8(5) | 11 | 11 | 33.5 | 30.5 |
| Gastrocnemius lateralis (R) | 37.73 | 7(0) | 8(5) | 9 | 12 | 27 | 38.5 |
| Gastrocnemius medialis (L) | 35.98 | 9(5) | 8(2) | 15 | 11 | **40*** | **28*** |
| Gastrocnemius medialis (R) | 38.51 | 8(4) | 9(2) | 11 | 12 | **43*** | **27*** |
| Gracilis (L) | 38.52 | 7(4) | 11(3) | 13 | 9 | 32 | 35 |
| Gracilis (R) | 37.51 | 8(4) | 10(3) | 12 | 11 | 33.5 | 32.5 |
| Peroneus (L) | 37.16 | 9(2) | 9(5) | 6.5 | 13 | 32 | 40 |
| Peroneus (R) | 36.44 | 8(2) | 9(4) | **6*** | **13*** | 32 | 33 |
| Sartorius (L) | 38.43 | 10(4) | 8(3) | 13 | 9 | 33.5 | 32.5 |
| Sartorius (R) | 39.38 | 9(3) | 9(4) | 11 | 13 | 29 | 35 |
| Semimembranosus (L) | 38.63 | 10(3) | 5(1) | 11 | 7 | 33.5 | 32 |
| Semimembranosus (R) | 38.13 | 10(1) | 5(4) | 7 | 13 | **29.5*** | **40*** |
| Soleus (L) | 37.34 | 10(4) | 7(3) | 13 | 11 | 34 | 29 |
| Soleus (R) | 38.18 | 8(3) | 9(4) | 13 | 11 | 43 | 29 |
| Semitendinosus (L) | 36.72 | 11(4) | 7(3) | 11 | 12 | 35 | 29 |
| Semitendinosus (R) | 34.86 | 12(2) | 6(5) | 7 | 12 | 32 | 35 |
| Tibialis anterior (L) | 39.43 | 11(2) | 7(5) | 9 | 13 | 29 | 37 |
| Tibialis anterior (R) | 38.62 | 11(3) | 7(4) | 9 | 13 | 32 | 37 |
| Tibialis posterior (L) | 40.29 | 7(2) | 11(5) | 11 | 12 | 32 | 33 |
| Tibialis posterior (R) | 39.92 | 7(1) | 11(6) | 7 | 14 | 32 | 35 |
| Vastus intermedialis (L) | 40.08 | 11(4) | 7(3) | 11 | 15 | 29 | 35 |
| Vastus intermedialis (R) | 40.61 | 10(5) | 8(2) | 11 | 13 | 29 | 37.5 |
| Vastus lateralis (L) | 41.46 | 8(4) | 10(3) | 11 | 12 | 28 | 36 |
| Vastus lateralis (R) | 42.72 | 9(5) | 9(2) | 11 | 13 | 32 | 35 |
| Vastus medialis (L) | 39.56 | 11(5) | 7(2) | 9 | 15 | **29*** | **40*** |
| Vastus medialis (R) | 42.06 | 12(5) | 5(2) | 11 | 15 | 30.5 | 40 |
| *denotes those with a significant difference (p<0.05) between high and low T2 values. Non remained significant after correction for multiple comparisons. | | | | | | | |
