# Supplementary material for: Water T2 could predict functional decline in patients with dysferlinopathy
Source: J Cachexia Sarcopenia Muscle. 2022 Sep 4;13(6):2888–97. doi: 10.1002/jcsm.13063 (PMC9745487; doi:10.1002/jcsm.13063)
Supplement: Supplementary file 4 — Table S2: Results of functional tests in patients with higher or lower than median T2 water value The table shows the results of the muscle function tests in the patients that were included in the higher or lower than median T2 water value on each muscle. N: number, smwt: 6 minutes walking test, m/s: meters per second, TTRW: time to run/walk 10 meters, TTUG: time to up&go test, [file JCSM-13-2888-s002.docx]

| muscle | Median water T2 value | n | NSAD score | | swmt (velocity m/s) | | TTRW (velocity TTRW/s) | | TTUG (velocity TTUG/s) | | Fat fraction (%) | | cCSA (cm^2^) | |
| --- | --- | --- | --- | --- | --- | --- | --- | --- | --- | --- | --- | --- | --- | --- |
|  |  |  | high T2 | low T2 | high T2 | low  T2 | high T2 | low T2 | high T2 | low T2 | high T2 | low  T2 | high  T2 | low  T2 |
| Adductor longus (L) | 38.49 | 13 | 45 | 35.5 | 1.51 | 1.01 | 0.19 | 0.14 | 0.15 | 0.12 | 20.48 | 20.48 | 147.40 | 151.13 |
| Adductor longus (R) | 39.86 | 17 | 33 | 41 | 1.03 | 1.42 | 0.15 | 0.21 | 0.11 | 0.14 | 14.01 | 16.64 | 119.30 | 161.49 |
| Adductor magnus (L) | 39.37 | 18 | 33 | 46 | 0.93 | 1.51 | 0.12 | 0.19 | 0.10 | 0.15 | 35.59 | 16.34 | 638.37 | 891.87 |
| Adductor magnus (R) | 39.00 | 18 | 33 | 45 | 1.12 | 1.13 | 0.13 | 0.15 | 0.13 | 0.14 | 26.63 | 39.59 | 1036.67 | 887.11 |
| Biceps femoris (L) | 39.64 | 17 | 34.5 | 44 | 1.11 | 1.41 | 0.15 | 0.16 | 0.13 | 0.14 | 17.29 | 31.62 | 843.03 | 882.01 |
| Biceps femoris (R) | 38.76 | 16 | 39 | 40.5 | 1.355 | 1.13 | 0.17 | 0.15 | 0.14 | 0.12 | 12.66 | 21.63 | 1196.40 | 855.21 |
| Extensor digitorum (L) | 40.53 | 18 | 39 | 35.5 | 1.355 | 0.98 | 0.17 | 0.15 | 0.14 | 0.10 | 15.34 | 14.39 | 318.80 | 337.40 |
| Extensor digitorum (R) | 39.64 | 18 | 35 | 44 | 1.13 | 1.03 | 0.15 | 0.15 | 0.14 | 0.10 | 15.65 | 14.03 | 334.85 | 328.26 |
| Gastrocnemius lateralis (L) | 36.90 | 16 | 40 | 33 | 1.08 | 1.21 | 0.16 | 0.12 | 0.13 | 0.12 | 12.29 | 30.83 | 576.05 | 112.29 |
| Gastrocnemius lateralis (R) | 37.73 | 15 | 44 | 35.5 | 1.41 | 1.03 | 0.18 | 0.13 | 0.14 | 0.12 | 12.17 | 28.92 | 143.19 | 144.57 |
| Gastrocnemius medialis (L) | 35.98 | 17 | 45 | 32.5 | 1.3 | 1.01 | 0.15 | 0.15 | 0.14 | 0.12 | 35.99 | 23.66 | **194.32*** | **121.60*** |
| Gastrocnemius medialis (R) | 38.51 | 17 | 35.5 | 46 | 1.13 | 1.51 | 0.15 | 0.19 | 0.12 | 0.14 | 69.14 | 21.67 | 198.53 | 127.30 |
| Gracilis (L) | 38.52 | 18 | **29**** | **45**** | 1.03 | 1.41 | 0.13 | 0.16 | 0.10 | 0.15 | 17.61 | 8.15 | 410.44 | 480.71 |
| Gracilis (R) | 37.51 | 18 | **32*** | **45.5*** | 1.11 | 1.32 | 0.13 | 0.17 | 0.12 | 0.14 | 14.58 | 13.20 | 412.43 | 405.77 |
| Peroneus (L) | 37.16 | 18 | 45 | 32 | 1.51 | 1.03 | **0.19*** | **0.11*** | **0.15*** | **0.10*** | 19.60 | 26.42 | **580.15*** | **215.23*** |
| Peroneus (R) | 36.44 | 17 | 45 | 32 | 1.495 | 0.93 | **0.18*** | **0.12*** | 0.15 | 0.10 | 8.49 | 31.86 | **560.70*** | **252.51*** |
| Sartorius (L) | 38.43 | 18 | 33.5 | 40.5 | 1.21 | 1.03 | 0.15 | 0.15 | 0.13 | 0.12 | 9.29 | 18.45 | 445.75 | 433.48 |
| Sartorius (R) | 39.38 | 18 | 33 | 45 | 1.12 | 1.13 | 0.14 | 0.15 | 0.13 | 0.14 | 9.18 | 17.62 | 455.72 | 362.92 |
| Semimembranosus (L) | 38.63 | 15 | 45 | 32 | 1.46 | 1.03 | 0.19 | 0.14 | 0.15 | 0.09 | 26.99 | 36.71 | 527.14 | 423.84 |
| Semimembranosus (R) | 38.13 | 15 | 44.5 | 36 | 1.46 | 0.92 | 0.19 | 0.11 | 0.15 | 0.10 | 15.71 | 41.76 | 494.11 | 473.31 |
| Soleus (L) | 37.34 | 17 | 40 | 34 | 1.075 | 1.30 | 0.16 | 0.13 | 0.11 | 0.14 | 32.66 | 19.41 | 1144.81 | 373.67 |
| Soleus (R) | 38.18 | 17 | 40 | 32 | 1.165 | 1.12 | 0.16 | 0.13 | 0.13 | 0.11 | 35.56 | 29.77 | **1504.21*** | **502.90*** |
| Semitendinosus (L) | 36.72 | 18 | 44 | 29 | 1.3 | 1.09 | 0.16 | 0.13 | 0.15 | 0.10 | 20.62 | 36.88 | **878.07**** | **225.65*** |
| Semitendinosus (R) | 34.86 | 18 | 40 | 28.5 | 1.36 | 1.01 | 0.17 | 0.13 | 0.14 | 0.10 | 24.38 | 35.51 | 689.02 | 293.40 |
| Tibialis anterior (L) | 39.43 | 18 | 44 | 35 | 1.41 | 0.92 | 0.18 | 0.12 | 0.14 | 0.10 | 10.03 | 16.97 | 524.02 | 516.54 |
| Tibialis anterior (R) | 38.62 | 18 | 44 | 31 | 1.13 | 1.12 | 0.16 | 0.13 | 0.15 | 0.11 | 18.28 | 17.69 | 596.67 | 352.93 |
| Tibialis posterior (L) | 40.29 | 18 | 33 | 36 | 1.12 | 1.13 | 0.16 | 0.15 | 0.13 | 0.14 | 21.81 | 12.90 | 422.53 | 476.18 |
| Tibialis posterior (R) | 39.92 | 18 | 33 | 45 | 1.09 | 1.30 | 0.15 | 0.15 | 0.13 | 0.14 | 22.46 | 11.82 | 474.29 | 458.18 |
| Vastus intermedialis (L) | 40.08 | 18 | 34 | 45 | 1.12 | 1.13 | 0.13 | 0.15 | 0.13 | 0.14 | 20.94 | 9.26 | 1653.70 | 1382.36 |
| Vastus intermedialis (R) | 40.61 | 18 | 32.5 | 44.5 | 1.105 | 1.27 | 0.13 | 0.16 | 0.12 | 0.14 | 21.78 | 23.01 | 1644.30 | 1552.22 |
| Vastus lateralis (L) | 41.46 | 18 | 33.5 | 40 | 1.21 | 1.11 | 0.15 | 0.15 | 0.13 | 0.12 | 15.19 | 10.67 | 1448.50 | 2037.58 |
| Vastus lateralis (R) | 42.72 | 18 | 33 | 45 | 1.12 | 1.41 | 0.12 | 0.16 | 0.13 | 0.14 | 24.99 | 7.51 | 1171.45 | 2023.66 |
| Vastus medialis (L) | 39.56 | 18 | 34 | 36 | 1.3 | 1.09 | 0.16 | 0.15 | 0.14 | 0.11 | 14.08 | 36.38 | 854.73 | 1060.89 |
| Vastus medialis (R) | 42.06 | 17 | 33.5 | 45 | 1.105 | 1.13 | 0.13 | 0.15 | 0.12 | 0.16 | 22.66 | 9.19 | 802.26 | 1654.79 |
| *p <0.05, **p <0.01. No values significant after correction for multiple comparisons. | | | | | | | | | | |  |  |  |  |
