# Supplementary material for: Water T2 could predict functional decline in patients with dysferlinopathy
Source: J Cachexia Sarcopenia Muscle. 2022 Sep 4;13(6):2888–97. doi: 10.1002/jcsm.13063 (PMC9745487; doi:10.1002/jcsm.13063)
Supplement: Supplementary file 5 — Table S3: Sensitivity and specificity of a T2H2O threshold in predicting decline in NSAD score in the initial and extension cohort [file JCSM-13-2888-s001.docx]

**Supplemental table 3:**

**Sensitivity and specificity of a T2_H2O_ threshold in predicting decline in NSAD score in the initial and extension cohort**

|  | |  | Decline of >5 points on NSAD in 3 years | | Decline of >1 point on NSAD in 1 year | |
| --- | --- | --- | --- | --- | --- | --- |
| **Muscle** | **Threshold** | **Analysis** | **Test cohort** | **Extension cohort** | **Test cohort** | **Extension cohort** |
| Adductor magnus | 39.0 ms | Sensitivity | 63% | 33% | 57% | 38% |
|  |  | Specificity | 78% | 90% | 71% | 100% |
| Vastus intermedius | 39.4 ms | Sensitivity | 100% | 33% | 86% | 25% |
|  |  | Specificity | 67% | 90% | 57% | 88% |
| Vastus lateralis | 40.5 ms | Sensitivity | 88% | 33% | 71% | 25% |
|  |  | Specificity | 78% | 70% | 71% | 63% |
| Vastus medialis | 40.1 ms | Sensitivity | 88% | 33% | 86% | 25% |
|  |  | Specificity | 67% | 80% | 57% | 75% |
| **Muscle grouping** |  |  |  |  |  |  |
| All four muscles over threshold bilaterally | Respective threshold for each muscle as above | Sensitivity | 63% | 17% | 43% | 13% |
|  |  | Specificity | 89% | 100% | 71% | 100% |
| At least one muscle over threshold bilaterally |  | Sensitivity | 100% | 50% | 86% | 50% |
|  |  | Specificity | 56% | 60% | 57% | 60% |
