# Supplementary material for: Water T2 could predict functional decline in patients with dysferlinopathy
Source: J Cachexia Sarcopenia Muscle. 2022 Sep 4;13(6):2888–97. doi: 10.1002/jcsm.13063 (PMC9745487; doi:10.1002/jcsm.13063)
Supplement: Supplementary file 6 — Data S1. Supporting Information [file JCSM-13-2888-s005.docx]

**Extension cohort**

**Supplemental methods:** **testing T2_H2O_ thresholds in the extension cohort**

In order to check if the results obtained in the Newcastle/Paris cohort could be extended to a larger number of patients, we selected a second cohort of 21 patients (extension cohort) that included ambulant patients who had a baseline NSAD score of more than 15 points and had completed baseline and year-3 NSAD assessments. This cohort included patients seen at Munich, Padova, Seville, Saint Louis, Tokyo and Washington DC. Functional assessments were performed at each site by local physiotherapists trained by the lead site physiotherapy team (Newcastle).

Data were acquired using 1.5 tesla clinical scanners in Padova (Siemens), Seville (Philips), Saint Louis (Siemens) and Washington DC (General Electric), and 3 tesla clinical scanners in Munich (Philips) and, Tokyo (Siemens). As T2 values from skeletal muscle scanned using 3 tesla scanners is reportedly about 10% lower than when scanned in 1.5 tesla scanners, a correction factor of 0.9 was applied to T2 values from the 1.5 tesla scanners ^1^.

The sensitivity and specificity of the threshold T2_H2O_ values identified in the Newcastle/Paris cohort were also assessed in the extension cohort. A true positive (TP) prediction occurred when T2_H2O_ was greater than the threshold on both sides and NSAD change was more negative than -5 points over three years. A false negative (FN) prediction occurred when the T2_H2O_ value was less than or equal to the threshold, but NSAD change was more negative than -5 points over three years. This process was repeated to determine if the thresholds were useful over a shorter period of one year. The same method of analysis was used for the one-year analysis, except that deterioration was defined as an NSAD change over one year that was greater than or equal to a 2-point decline, as this is greater than the 1.68 annual progression expected in the cohort and NSAD can only change by integers from one year to the next.

**Supplemental results:**

**Demographic data**

The extension cohort consisted of 16 ambulant patients (8 male) assessed in Munich (1), Padova (4), Seville (3), Saint Louis (4), Tokyo (2) or Washington DC (2). Patients had a median age of 33.5 years (range 11-50 years) and had had symptoms for a median of 11 years (range 3-23 years).

**Testing threshold in the extension cohort**

The sensitivity of predicting decline on the NSAD score with the T2_H2O_ thresholds over both one and three was higher in the initial cohort than in the extension cohort over both one and three years (*Supplemental figure 2 and supplemental table 3*).

1. Gold GE, Han E, Stainsby J, Wright G, Brittain J, Beaulieu C. Musculoskeletal MRI at 3.0 T: relaxation times and image contrast. AJR Am J Roentgenol 2004;183:343-351.
